# Phenotypic variation of a new synthetic allotetraploid *Arabidopsis kamchatica* enhanced in natural environment

**DOI:** 10.3389/fpls.2022.1058522

**Published:** 2023-01-04

**Authors:** Rie Shimizu-Inatsugi, Aki Morishima, Beatriz Mourato, Kentaro K. Shimizu, Yasuhiro Sato

**Affiliations:** ^1^Department of Evolutionary Biology and Environmental Studies, University of Zurich, Zurich, Switzerland; ^2^Kihara Institute for Biological Research, Yokohama City University, Yokohama, Japan

**Keywords:** polyploid, neopolyploid, phenotypic variation, vegetative organ, *in natura*, *Arabidopsis*

## Abstract

The phenotypic variation of vegetative organs and reproductive organs of newly synthesized and natural *Arabidopsis kamchatica* genotypes was investigated in both a controlled environment and a natural environment in an experimental garden. When we compared the variation of their leaf shape as a vegetative organ, the synthetic *A. kamchatica* individuals grown in the garden showed larger variation compared with the individuals incubated in a growth chamber, suggesting enhanced phenotypic variation in a natural fluctuating environment. In contrast, the natural *A. kamchatica* genotypes did not show significant change in variation by growth condition. The phenotypic variation of floral organs by growth condition was much smaller in both synthetic and natural *A. kamchatica* genotypes, and the difference in variation width between the growth chamber and the garden was not significant in each genotype as well as among genotypes. The higher phenotypic variation in synthetic leaf may imply flexible transcriptomic regulation of a newly synthesized polyploid compared with a natural polyploid.

## Introduction

Allopolyploid species have been considered to possess higher intraspecific phenotypic diversity, as a nature of its merged genomes from two closely relative but distinct species (Paterson, 2005; Van de Peer et al., 2017). The allopolyploid genome can be considered as a permanent heterozygote in this sense. In fact, many agriculturally or ecologically important polyploid plants are reported to have transgressive, intermediate, or wider phenotypes beyond progenitors (Comai, 2005; Gallego-Tévar et al., 2018; Shimizu, 2022). For this reason, polyploidization has been considered as one of the most important motive forces for evolution by expanding phenotypic diversity. However, polyploidization accompanies a genetic bottleneck due to the limited number of progenitors involved in the establishment of a polyploid population. Thus, the genetic variation could have only limited contribution to the phenotypic diversity of a newly synthesized polyploid group. Even if the genetic diversity will be restored by introgression, multiple origins, and new mutations gradually (Shimizu-Inatsugi et al., 2009; Novikova et al., 2017; Paape et al., 2018), the newly synthesized polyploid should cope with the reduced genetic diversity for speciation. Thus, in addition to genetic diversity, phenotypic variation within the same genotype would be more important at the beginning of a new polyploid group. The intraspecific phenotypic diversity might also contribute to widen the habitat range of a species (Westerband et al., 2021), most probably by higher adaptive ability for dispersal. In fact, some invasive species have been found to have higher phenotypic diversity than natives (Funk, 2008; Hiatt and Flory, 2020). It is also consistent with the theoretically expected wider distribution of polyploid species or the distribution at extreme environments (Rice et al., 2019), assuming that polyploid had higher phenotypic diversity.

Several factors can cause phenotypic diversity within the same genotype. Developmental noise in ontogeny would be an important factor to realize the phenotypic diversity within the same genotype, which could also be stimulated by environmental factors (Westerband et al., 2021). In addition, epigenetic diversity may be another important factor for phenotypic diversity, which is also supported by a previous study showing phenotypic disturbance in the mutant line lacking DNA methylase (Kakutani et al., 1996) as well as by famous examples of phenotypic variation caused by single epialleles in floral symmetry of antirrhinum (Cubas et al., 1999) or fruit ripening (Manning et al., 2006). At the early stage of polyploid species with reduced genetic diversity, epigenetic diversity produced by the genome merge would play a crucial role to generate high phenotypic diversity as reviewed by Shimizu (2022).

In this study, we quantitatively investigated the difference in phenotypic variation between new and established polyploids and statistically evaluated how the environment can influence their variation. We regenerated a new allotetraploid by crossing *A. halleri* subsp. *gemmifera* and *A. lyrata* subsp. *petraea* mimicking the natural allotetraploid species *A. kamchatica.* By using selfing lines, we can focus on phenotypic variation within the same genotype. The natural and synthetic *A. kamchatica* were incubated in continuous and stable conditions in a growth chamber and in a fluctuating natural environment in an experimental garden. The range of phenotypic diversity between synthetic and established lines as well as the change of that range according to environments are compared by examining two hypotheses: 1) Does the range of phenotypic variation differ between new and established polyploids? 2) Is the phenotypic variation higher in naturally fluctuating environments than in regulated chambers? At last, we will discuss the evolutionary significance of polyploid phenotypic variation.

## Materials and methods

### Plant incubation

We incubated two natural *A. kamchatica* lines, from Alaska, US (ALK, GPS coordinates: 63.42, 145.84) and Murodo, Japan (MRD, GPS coordinates: 36.58, 137.60), and two synthetic *A. kamchatica* lines (RS2 and RS7). The natural lines were self-fertilized for at least twice in the laboratory after the collection from natural populations. Considering that they are selfing species, additional selfings in the lab should have made them highly homogeneous. The synthetic lines are produced by crossing *A. halleri* subsp. *gemmifera* (Japan) (maternal, GPS coordinates: 35.10, 134.92) and *A. lyrata* subsp. *petraea* (Russia, GPS coordinates: 68.8, 160.3) (paternal). Both of the diploid lines are also nearly homogenous because the former was repeatedly self-fertilized by bud pollination and the latter is originally self-compatible and self-fertilized twice in the laboratory (Briskine et al., 2017; Paape et al., 2018). The F1 hybrid diploid individuals were polyploidized by colchicine treatment on seedling (RS2) or autonomously without colchicine treatment (RS7), and the second generation of a selfed tetraploid offspring was used for this experiment, which also made these lines highly homogeneous. They were incubated either in a growth chamber (22˚C / 20˚C, 16 h light/8 h dark, 60% relative humidity) or in the experimental garden located at the Irchel campus of University of Zurich (Zurich, Switzerland) in 2015 and 2016, independently. For the garden experiment, the plants were first incubated in the chamber for 2–3 weeks and then transplanted to the garden plot in November to be incubated there until July the next year.

### Leaf and flower collection and measurement

**Figure 1:**
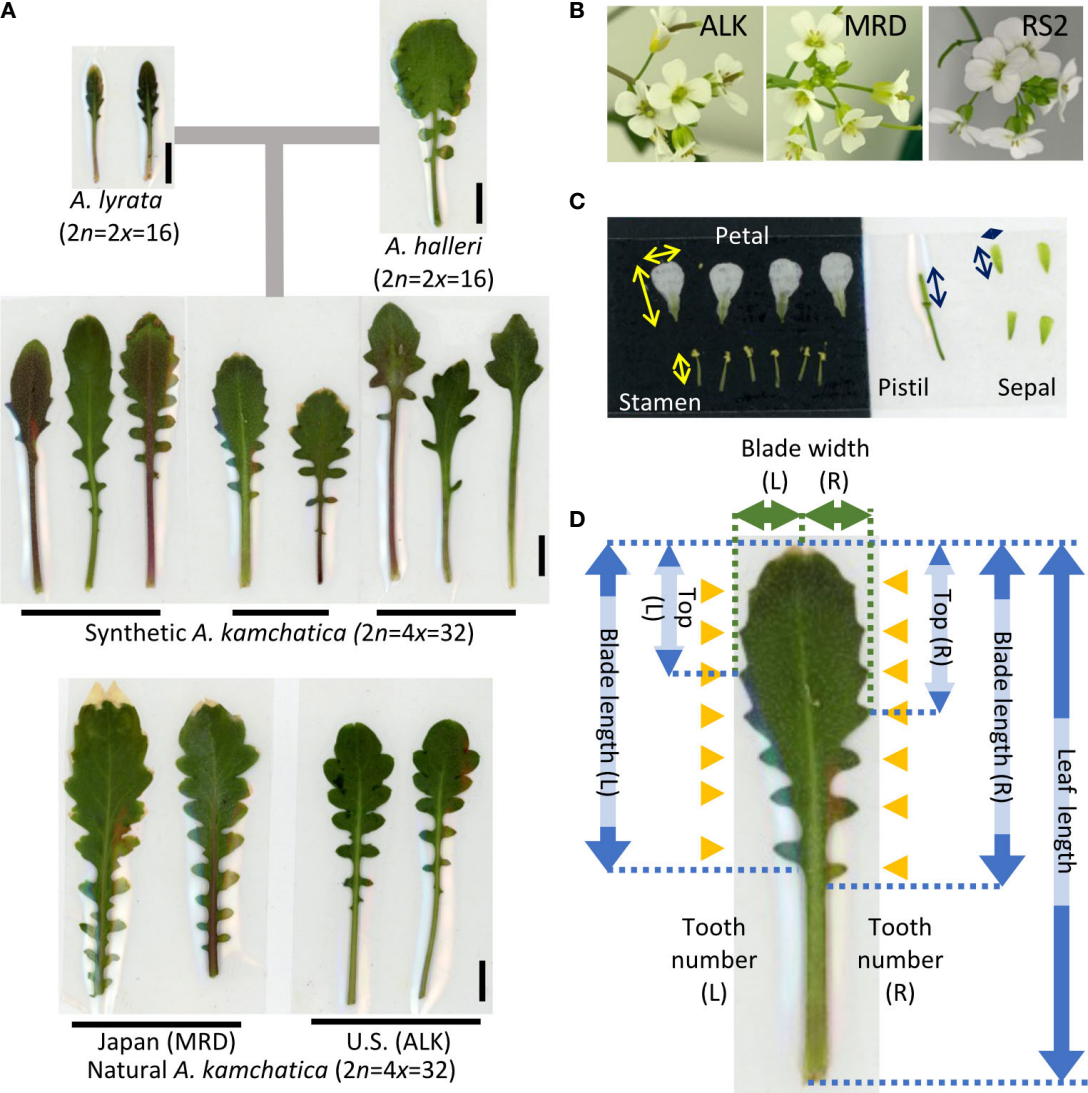
Representative leaf and flower shape of *A. kamchatica* and the traits used for the analysis. **(A)** Representative leaves of diploid progenitors, and synthetic and natural *A. kamchatica* genotypes grown in the chamber. The horizontal lines suggest the leaves collected from the same individual. The vertical lines indicate 1 cm. **(B)** Representative flowers of *A. kamchatica*, ALK, MRD and RS2. **(C)** An example of pressed floral organs. The arrows indicate the measured positions of each organ, vertical (length) and horizontal (width). **(D)** The positions of measured traits on leaf morphology. The arrows indicate the length or width of each trait, and the orange triangles indicate the position of leaf tooth on R (right) and L (left) sides, respectively.

The leaf samples (except for ALK in 2015 in the garden) and flower samples (except for 2015 in the garden) were collected soon after they started flowering. It was about 4 months after the germination (including 6 weeks of vernalization at 4˚C as seedlings) for the chamber individuals and from February to June for the garden individuals. We selected mature leaves and fully and newly open flowers for sampling. The sample sizes (summarized in **Supplementary Table S1**) were unbalanced among lines (5 to 104 in leaf and 7 to 79 in flower) due to the high variation of growth speed as well as flowering period among lines. Leaves were pressed and fixed on a paper by transparent sticky tape immediately after the collection (as seen in **Figure 1A**). Flowers were immediately dissected into each organ, and all parts were pressed and fixed on a paper by transparent sticky tape (as seen in **Figure 1B**). Each trait was measured on the specimen using a digital caliper. The detailed position of each trait is shown in **Figures 1B, C** (flower) and **Figure 1D** (leaf). We counted the number of each organ in one flower. For petals and sepals, we measured the length and width and measured the length of stamens and pistils. Blade length represents the distance from the tip of the blade to the bottom of the last lobe. Top represents the distance from the tip of the blade to the widest point of the blade. These traits as well as blade width were independently measured on the right and left sides, and thus two values of R (right) and L (left) are available.

To calculate the ratio of the two factors, we first calculated the ratio of each leaf/side to take the average of all replicates. To calculate the leaf shape uniformity on R and L, we first took the difference between the two values (R and L) to calculate the difference from their mid-value in percent figures and then took the average among replicates.

All the visualization and statistical analyses were performed using R version 4.0.3 (R Core Team, 2020). The coefficient of variation (i.e., CV = standard deviation/average) was first calculated as an index of phenotypic variation. To statistically test whether the phenotypic variation in each trait was significantly different among the genotypes (comparing four genotypes in the same growth condition) or among the growth conditions (comparing the same genotype between two or among three growth conditions), we then used the Levene test to compare variance among the groups. The null hypothesis of the Levene test was equal phenotypic variation among the groups. The alternative hypothesis indicated statistically significant differences of phenotypic variation among the groups. To make the Levene test robust against the distribution of each phenotype, we combined Brown–Forsythe tests for the group median (Brown and Forsythe, 1974) with zero correction (Noguchi and Gel, 2010). *P*-values were determined by 1,000-times bootstraps to deal with the unbalanced sample size. To perform this robust-type Levene test, we used the levene.test function of the lawtest package (Gastwirth et al., 2020) implemented in R. The result of the Levene test is summarized in **Supplementary Tables S2**, **S3** as the category of *P*-values (*P* < 0.05, *P* < 0.01, *P* < 0.001), and the result among genotypes is referred in text as needed.

## Results

### Leaf morphology

**Figure 2:**
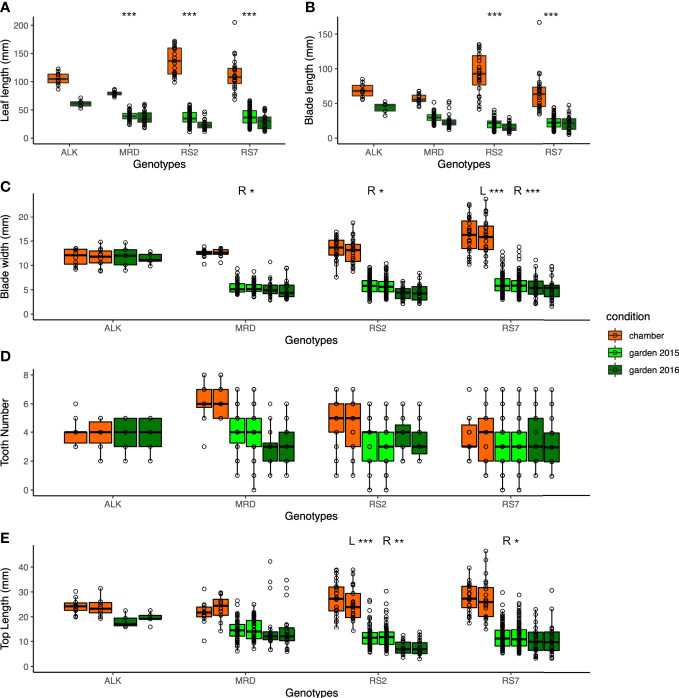
The traits representing the size and the shape of leaves. **(A)** Leaf length (mm). **(B)** Blade length (mm). **(C)** Blade width (mm). **(D)** Tooth number. **(E)** Top length (mm). Two neighboring columns in the same color in **(C–E)** represent the values of the left side (left) and right side (right) of leaves. The number of asterisks above the columns represents the category of *P* values (*P < 0.05; **P < 0.01; ***P < 0.001) by Levene test to examine the statistical significance of variation in each trait among growth conditions. When the test is conducted independently on the left and right sides, the result is shown as L and R.

We first compared the traits that were directly measured, i.e., leaf size and shape, of each genotype in different growth conditions, i.e., in a growth chamber and in an experimental garden, for two seasons of 2015 and 2016. As many of the leaves were not perfectly symmetrical, we measured some traits independently on both the right side (R) and left side (L). Generally, the leaf lengths of all genotypes of synthetic and natural lines are longer in the chamber compared with those in the garden (**Figure 2A**). A similar trend was observed in the blade length and blade width except for ALK (**Figures 2B, C**), suggesting the harsher condition of the garden generally making the leaf size smaller. We also evaluated the statistical significance of the width of variation by Levene test whether each genotype shows different phenotypic variation between the growth conditions, except for ALK due to its small sample size as well as lack of incubation in the garden in 2015. The other three genotypes showed high significance in leaf length, and two synthetic lines but not MRD showed significant difference between the growth conditions when the blade length and blade width on one or both sides were analyzed (**Table S2**).

As a proxy of the complexity of the pinnately lobed leaf, we counted the number of tooth points on R and L of the leaf blade, representing the lobe number. Compared with the blade size change, the tooth number on each blade showed smaller change except for a few lines. In the chamber, the tooth number was generally similar between R and L in natural lines but the synthetic lines had a slightly wider variation (**Figure 2D**). The variation became slightly greater in natural lines in the garden in 2015 and 2016, but no statistical significance was detected by Levene test (**Table S2**). Another trait representing the leaf complexity, the length from the tip to the widest point of each blade (top length), showed a large decrease in the garden than in the chamber in synthetic lines, but not in ALK and MRD (**Figure 2E**).

In all leaf morphology traits, the synthetic lines generally showed a larger decrease in the garden compared with the chamber. In addition, the range of each trait as calculated by CV value was larger in the garden than in the chamber (**Table 1**) in most traits and genotypes, suggesting the larger variation of phenotype. Levene test also supported these differences in phenotypic variation between the growth conditions for many of the traits (**Table S2**). In summary, among the four genotypes, two synthetic lines showed larger changes and variations against growth conditions.

**Table 1:** CV values of each leaf trait.

Genotype	Incubation	Leaf length	Blade length	Blade width		Tooth number		Top length	
				Left	Right	Left	Right	Left	Right
ALK	Chamber	0.11	0.15	0.15	0.17	0.24	0.25	0.13	0.15
ALK	2016 garden	0.11	0.19	0.20	0.10	0.34	0.34	0.15	0.12
MRD	Chamber	0.05	0.13	0.07	0.07	0.23	0.18	0.23	0.21
MRD	2015 garden	0.19	0.22	0.23	0.21	0.31	0.38	0.29	0.30
MRD	2016 garden	0.35	0.45	0.35	0.38	0.46	0.42	0.62	0.56
RS2	Chamber	0.18	0.31	0.17	0.20	0.37	0.37	0.25	0.27
RS2	2015 garden	0.35	0.33	0.28	0.28	0.39	0.43	0.36	0.37
RS2	2016 garden	0.38	0.43	0.32	0.40	0.34	0.38	0.35	0.40
RS7	Chamber	0.26	0.44	0.23	0.23	0.39	0.48	0.21	0.30
RS7	2015 garden	0.36	0.36	0.32	0.33	0.48	0.46	0.39	0.42
RS7	2016 garden	0.45	0.54	0.42	0.44	0.49	0.52	0.53	0.62

### Leaf shape balance on right and left sides

**Figure 3:**
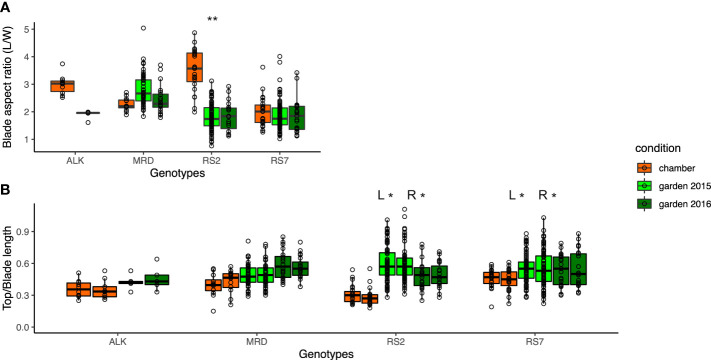
The traits representing the balance of the blade shape. **(A)** Blade aspect ratio is calculated by dividing the blade length by the sum of the left- and right-blade width. **(B)** Ratio of top length is calculated by dividing the top length by the blade length, representing the relative size of the upper round part of the leaf. The asterisk represents the P-value by Levene test (*: P<0.05; **: P<0.01; ***: P<0.001).

As the measured values of leaf length and width turned out to be vulnerable to the environment, we investigated the aspect ratio and right-and-left balance of the blade as proxies of leaf shape variation. We calculated the aspect ratio of the blade (blade length/sum of right and left width) (**Figure 3A**) as well as the top and blade length ratio for each right and left sides (**Figure 3B**). While ALK and RS2 showed a decrease in blade aspect ratio in the garden, MRD and RS7 did not show a big change in the value. The top and blade length ratio changed drastically only in RS2 but not in the other three genotypes. In the sense of phenotypic variation between the growth conditions, Levene test detected a significant difference in variation for the blade aspect ratio only in RS2 and for the top and blade length ratio in both RS2 and RS7. These results suggest the wider phenotypic variation of leaf morphology in synthetic lines in the garden than in the chamber, whereas that of natural lines was similar between garden and chamber. On the other hand, the variation among the genotypes showed a significant difference in chamber and garden 2015 conditions in many traits (**Table S2**), supporting the difference among the genotypes.

We also investigated the leaf shape unevenness on the right side and left side by calculating the difference (%) of R and L of each factor in each leaf (**Table 2**), in which the larger number suggests the larger imbalance between the right and left sides. The unevenness in blade width, tooth number, top length, and blade W/L ratio was higher in synthetic lines than in natural ones in the chamber. The unevenness was slightly enhanced in natural lines, but not in the synthetic lines in the garden. This result suggests that the leaf shape unevenness of right and left sides in synthetic lines is high regardless of environment and that of natural lines is low in stable conditions but can be enhanced by fluctuating conditions.

**Table 2:** The unevenness of the right side and left side of a leaf.

		Blade width			Tooth number			Top			Blade length/width		
Genotype	Incubation	Average (%)		SD	Average (%)		SD	Average (%)		SD	Average (%)		SD
ALK	Chamber	1.33	±	0.89	2.01	±	3.51	2.32	±	1.23	1.33	±	0.89
ALK	2016 garden	3.41	±	2.12	4.00	±	5.48	1.79	±	2.24	3.41	±	2.12
MRD	Chamber	0.86	±	0.70	1.98	±	3.72	4.22	±	3.74	0.86	±	0.70
MRD	2015 garden	2.43	±	1.87	5.10	±	9.05	3.77	±	3.00	2.43	±	1.87
MRD	2016 garden	4.97	±	4.47	7.24	±	9.63	4.31	±	4.38	4.97	±	4.47
RS2	Chamber	3.36	±	3.62	3.96	±	5.41	4.52	±	4.09	3.36	±	3.62
RS2	2015 garden	2.87	±	2.39	5.97	±	10.27	4.28	±	3.85	2.87	±	2.39
RS2	2016 garden	3.94	±	3.50	3.58	±	4.09	2.24	±	1.99	3.94	±	3.50
RS7	Chamber	2.21	±	1.77	6.75	±	6.40	4.40	±	2.95	2.21	±	1.77
RS7	2015 garden	2.34	±	2.00	5.40	±	10.53	4.48	±	3.82	2.34	±	2.00
RS7	2016 garden	2.82	±	2.09	5.08	±	6.54	2.91	±	2.78	2.82	±	2.09

### Flower morphology

In comparison with the shape of leaf as a vegetative organ, we also analyzed the phenotypic variation of flower as a reproductive organ. The number and size of four floral organs (sepal, petal, stamen, and pistil) were measured after dissection. The number of each organ was largely stable in both chamber and garden except for few flowers (**Table 3**). The variation was found only in the sepal and petal numbers of synthetic lines in chamber, and the stamen number was the most variable found in all genotypes in either or both chamber and garden. We did not find any variation of pistil number in any lines and conditions. Overall, the variation in floral organ number was very limited, suggesting the robustness in flower whorl development against external stimuli. The variations of floral organ numbers were not significant among the four genotypes grown in the same condition (chamber or garden) (Levene test, *p* > 0.05), except for the stamen number among four genotypes in chamber (*p* < 0.001).

**Table 3:** The number of floral organs.

		Sepal			Petal			Stamen		
Genotype	Incubation	Average (%)		SD	Average (%)		SD	Average (%)		SD
ALK	Chamber	4.00	±	0.00	4.00	±	0.00	5.98	±	0.16
ALK	2016 garden	4.00	±	0.00	4.00	±	0.00	6.00	±	0.00
MRD	Chamber	4.00	±	0.00	4.00	±	0.00	6.00	±	0.00
MRD	2016 garden	4.00	±	0.00	4.00	±	0.00	5.88	±	0.35
RS2	Chamber	3.97	±	0.16	3.96	±	0.19	5.73	±	0.55
RS2	2016 garden	4.00	±	0.00	4.00	±	0.00	6.00	±	0.00
RS7	Chamber	4.05	±	0.28	4.04	±	0.41	6.01	±	0.38
RS7	2016 garden	4.00	±	0.00	4.00	±	0.00	5.89	±	0.33

**Figure 4:**
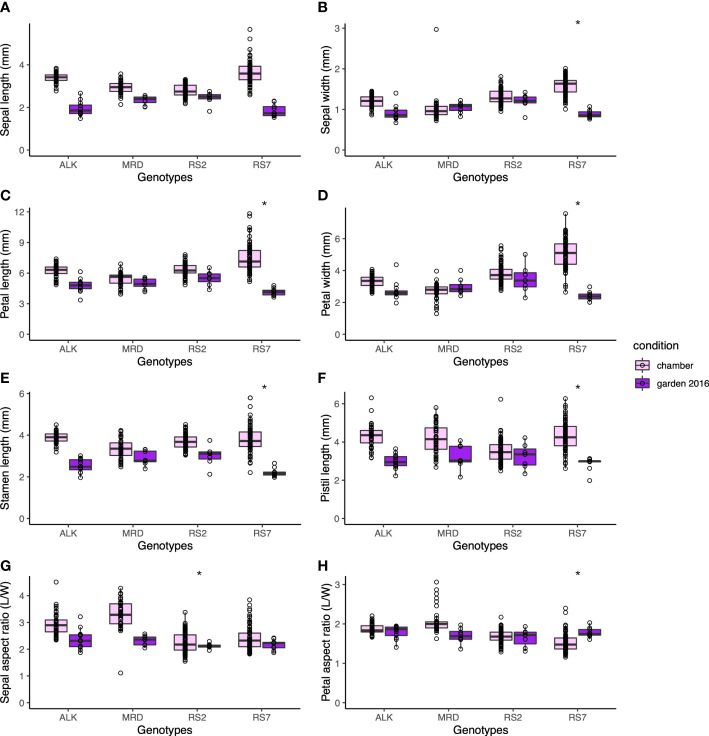
The traits representing the size and shape of floral organs. Sepal length **(A)** and width **(B)**. Petal length **(C)** and width **(D)**. **(E)** Stamen length. **(F)** Pistil length. **(G)** Sepal aspect ratio, length divided by width. **(H)** Petal aspect ratio, length divided by width. The asterisk represents the P-value by Levene test (*: P<0.05; **: P<0.01; ***: P<0.001).

The size of each organ tends to be smaller in the garden than in the chamber. Compared with leaf size, which could be less than half in the garden than in the chamber, the decrease in floral organ size was smaller. The petal length and width, which might reflect the flower size the best, decreased most drastically in RS7 but less in RS2 and natural lines (**Figures 4A–D**). A similar level of decrease was found in stamen and pistil sizes (**Figures 4E, F**). The decrease in their size was slightly smaller in MRD and RS2 than in ALK and RS7, but statistical significance was only detected in most organs in RS7. These results suggest that the effect of the environment on floral organ shape in each genotype was smaller than that on leaf shape. In other words, floral organ shape had higher stability against external stimuli, and a small variation in phenotypic plasticity among conditions was found only in RS7 (**Table S3**, *p* < 0.05).

Consistent with the robustness of length of each factor, the aspect ratios of sepal and petal were also stable among genotypes and conditions (**Figures 4G, H**). Compared with the sepal aspect ratio for which natural lines showed a slight change in the two conditions, the petal aspect ratio was very similar to each other in all genotypes and conditions. Levene test detected the significant difference in variation between conditions only in the sepal aspect ratio of RS2 (**Table S3**, *p* < 0.05). This result means that even if the ultimate size of the floral organs changes according to conditions, their shape hardly changes.

### Size balance between reproductive organs

**Figure 5:**
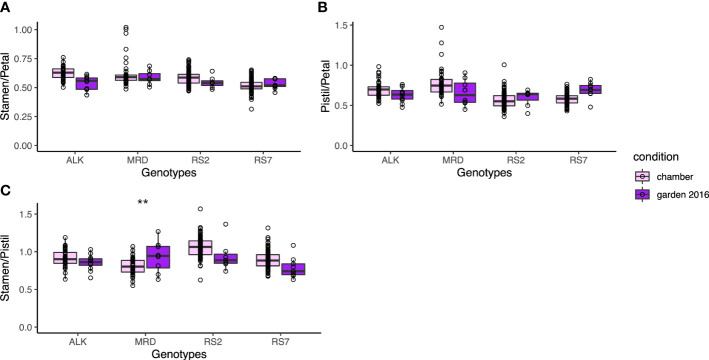
The traits representing the relative position of each organ. **(A)** Ratio of stamen length and petal length. **(B)** Ratio of pistil length and petal length. **(C)** Ratio of stamen length and pistil length. The asterisk represents the P-value by Levene test (*: P<0.05; **: P<0.01; ***: P<0.001).

We next focused on the relative size between floral organs, as it may affect the auto-pollination of *A. kamchatica* as a selfing species. We calculated three indices of the relative length, stamen/petal, pistil/petal, and stamen/pistil, depending on their lengths. All indices did not show a significant difference among genotypes and between conditions (**Figure 5**). It suggests that the relative positions of these organs were maintained in spite of the size variation of these organs. In addition, the variations of each index did not have a significant difference in variation between conditions except for the stamen/pistil of MRD (*p* < 0.001), in which the variation was bigger in the garden. Nevertheless, the morphology of floral organs was much less variable among genotypes and between conditions compared with the leaf shape (**Figure 3**), and most of all, the phenotypic variation in the same genotype was not affected by growth condition. This result implies the developmental robustness of the morphology of floral organs by strict genetic control behind.

## Discussion

### Phenotypic variation of vegetative organs larger in a natural environment than in a chamber

In this study, we investigated the width of phenotypic variation of both leaves and flowers, using two natural and two synthetic genotypes of allotetraploid species *A. kamchatica*. We examined the difference of phenotypic variation in constant growth conditions in a chamber and in naturally fluctuating conditions in an experimental garden. Floral organs showed similar sizes and shapes between conditions as well as among genotypes (**Figure 4**). In addition, the width of phenotypic variation between two conditions was not significantly different in most traits in both natural and synthetic lines (**Table S2**). In contrast, a wider range of organ size as well as organ shape was found in leaves among genotypes and conditions, which we could detect as larger values of CV in leaf (**Table 1**) than those in flower (**Table 4**). Especially, the phenotypic variation of many leaf shape traits was amplified by a natural condition, detected as much lower *p*-values by Levene test among growth conditions in synthetic genotypes than natural genotypes (**Figure 2** and **Table S2**).

**Table 4:** CV values of each flower trait.

Genotype	Incubation	Sepal length	Sepal width	Petal length	Petal width	Stamen length	Pistil length
ALK	Chamber	0.07	0.12	0.10	0.11	0.07	0.14
ALK	2016 garden	0.17	0.20	0.14	0.22	0.13	0.13
MRD	Chamber	0.10	0.34	0.13	0.19	0.14	0.19
MRD	2016 garden	0.09	0.12	0.10	0.17	0.12	0.19
RS2	Chamber	0.10	0.14	0.10	0.16	0.09	0.17
RS2	2016 garden	0.12	0.17	0.13	0.25	0.17	0.20
RS7	Chamber	0.15	0.14	0.18	0.18	0.17	0.17
RS7	2016 garden	0.16	0.11	0.09	0.13	0.10	0.13

Furthermore, even when significant variation was found in the actual size of floral organ among genotypes or conditions, the traits representing the relative length between organs or the shape of each organ showed more uniform values among genotypes. The relative length of stamens and a pistil was stable around 1.0 without significant difference among genotypes and conditions (**Figure 5**), suggesting the importance of keeping their ratio close for successful self-pollination. On the other hand, the petal aspect ratio was also one of the least variable traits among genotypes. The fact that the flower sizes of natural allotetraploid with old origin of approximately 0.1 M years old (Tsuchimatsu et al., 2012; Paape et al., 2018) and synthetic tetraploid with new origin are very similar may also support the importance of outcrossing, instead of flower downsizing by selfing syndrome. Assuming that the flower shape is most largely influenced by petal size and shape, this may imply the significance of outcrossing even in this self-compatible species.

In previous studies, large intraspecific phenotypic variation of flower tended to attract more interest of studies and thus many reports about outstanding phenotypic variations, e.g., dimorphism (Potente et al., 2022), color shift (Gómez et al., 2020), and impact of environment on phenotype (March-Salas et al., 2021), have been published. In contrast, reports dealing with the lack of phenotypic variation can be barely found. Yet, in some species, lack of phenotypic variation in reproductive organs might also represent an important significance in evolution, reflecting some selective pressure.

While leaf morphology showed generally larger variations in size and shape than floral organs among genotypes as well as conditions, the synthetic lines tend to have larger variations in most traits. Most importantly, the range of phenotypic variation of synthetic lines but not natural lines was amplified by fluctuating conditions in the garden, in spite of the lack of genetic variation among individuals. In the previous study evaluating phenotypic plasticity of worldwide genotype collection of allopolyploid and diploid wild strawberry, no difference in plasticity was found between ploidy levels (Wei et al., 2019). These results may suggest that established polyploid species reduce their plasticity at the early step of “diploidization of polyploid” or adaptation. Our study further adds an insight about the difference between established and new polyploids, showing higher phenotypic variation of new polyploids than established polyploids. When we compared the balance of size between right and left sides of a leaf, the unevenness of synthetic lines was only slightly larger than that of the natural lines (**Table 2**), suggesting that the symmetrical development is still under some regulation if not perfect. This suggests that these variations in synthetic *A. kamchatica* did not occur stochastically as evoked by the idea of “genome shock” by polyploidization (Bird et al., 2018).

### How does new polyploid show larger phenotypic variation, and can it be advantageous?

Although generally large phenotypic variation or instability of newly synthesized polyploid has been recognized and reported so far (Madlung et al., 2002; Chen, 2007), this is the first study to compare the effect of the environment to examine the magnifying effect of the fluctuating environment on the width of variation. Although some previous papers report large polyploid variation in controlled conditions, the variation might have been even larger if they had been conducted in the wild environment.

The genetic variation in natural lines should be low due to multiple times self-fertilization in the lab, and that of synthetic lines should be even lower, theoretically null, due to their diploid hybrid origins before polyploidization. In spite of lower genetic variation, both of the two synthetic lines showed significantly larger variation in most leaf phenotypic traits than natural lines. These high phenotypic variations may be attributed to the variation of the epigenetic status (Chen, 2007; Schmid et al., 2018). Allopolyploidization is known to change the progenitors’ patterns of DNA methylation, generating a new pattern in early generations of allopolyploids (Yuan et al., 2020; Jiang et al., 2021). Even though the direct effect of DNA methylome variation on phenotype (Seymour and Becker, 2017) as well as the effect of a fluctuating environment on DNA methylation is not clear yet, our results imply that a fluctuating environment may stimulate the width of epigenetic status.

Considering the fact that a polyploidization event changes the epigenetic patterns of progenitors genomes in the new polyploid genome, this enhancement of variation should be derived from the wider transcriptomic pattern of neopolyploids in natural condition. This phenotypic enhancement could contribute to the adaptation of neopolyploids. Indeed, in our preliminary analysis of transcriptomic patterns of new synthetic polyploids of *A. kamchatica*, a larger variation among biological triplicates compared with natural polyploids has been observed (unpublished data), which is consistent with this idea. Polyploidization usually works as a genetic bottleneck in evolution, as only a limited number and genotype of progenitors contribute to the establishment of a new polyploid species (Shimizu-Inatsugi et al., 2009; Novikova et al., 2017). Nevertheless, polyploidization was considered as a motive force in evolution. A higher phenotypic variation of a new polyploid might contribute to solve this contradiction.

After polyploidization, offspring of early generations may undergo adaptation to new habitats. As a consequence, the epigenetic status should be stabilized according to time. This might be the reason we detected less phenotypic variation among conditions in established natural genotypes in this study or in a previous report (Wei et al., 2019). In addition, the difference in the reaction norm of our two natural populations could be attributed to their distinct adaptation measures to distinct habitats, ALK from a high-latitude population in Alaska and MRD from a low-latitude but high-altitude population in Japan.

In conclusion, we propose the positive effect of a fluctuating environment on the wider possibility of phenotypic diversification in polyploid species. The higher phenotypic variation of the polyploid in nature at the early stage of speciation may provide higher chances of adaptation to a wider habitat, assuming that epigenetic variation can be inherited. They will undergo natural selection in each habitat, and their epigenetic status will get stabilized again in the course of time, expanding the habitat to a wider (Akiyama et al., 2021) or even harsher environment (Rice et al., 2019) than progenitors. They will also obtain their own genetic variation advantageous in each habitat, but the speed of genetic evolution is much slower than that of epigenetic evolution, and thus epigenetic variation may be a major contributing factor at the early stage of speciation. The epigenetic variation among isogenic individuals right after polyploidization as well as its significance on phenotypes should be addressed in future studies. In addition, the investigation of not only morphological but also physiological variation would be needed to further understand the significance of polyploidy in evolution.

## Data availability statement

The original contributions presented in the study are included in the article/**Supplementary Material**. Further inquiries can be directed to the corresponding author.

## Author contributions

RS-I planned the experiment. AM and BM acquired the phenotypic data. RS-I and YS analyzed the data. KS, YS, and RS-I wrote the manuscript. All authors contributed to the article and approved the submitted version.

## References

[B1] Akiyama R. Sun J. Hatakeyama M. Lischer H. E.L. Briskine R. V. Hay A.. (2021). Fine-scale empirical data on niche divergence and homeolog expression patterns in an allopolyploid and its diploid progenitor species. New Phytol. 229 (6), 3587–3601. doi: 10.1111/nph.17101 33222195 PMC7986779

[B2] Bird K. A. VanBuren R. Puzey J. R. Edger P. P. (2018). The causes and consequences of subgenome dominance in hybrids and recent polyploids. New Phytologist. 220 (1), 87–93. doi: 10.1111/nph.15256 29882360

[B3] Briskine R. V. Paape T. Shimizu-Inatsugi R. Nishiyama T. Akama S. Sese J.. (2017). Genome assembly and annotation of arabidopsis halleri, a model for heavy metal hyperaccumulation and evolutionary ecology. Mol. Ecol. Resour. 17 (5), 1025–1036. doi: 10.1111/1755-0998.12604 27671113

[B4] Brown M. B. Forsythe A. B. (1974). Robust tests for the equality of variances. J. Am. Stat. Assoc. 69 (346), 364–367. doi: 10.1080/01621459.1974.10482955

[B5] Chen Z. J. (2007). Genetic and epigenetic mechanisms for gene expression and phenotypic variation in plant polyploids. Annu. Rev. Plant Biol 58, 377–406. doi: 10.1146/annurev.arplant.58.032806.103835 17280525 PMC1949485

[B6] Comai L. (2005). The advantages and disadvantages of being polyploid. Nat. Rev. Genet 6, 836–846. doi: 10.1038/nrg1711 16304599

[B7] Cubas P. Vincent C. Coen E. (1999). An epigenetic mutation responsible for natural variation in floral symmetry. Nature. 401 (6749), 157–161. doi: 10.1038/43657 10490023

[B8] Funk J. L. (2008). Differences in plasticity between invasive and native plants from a low resource environment. J. Ecol. 96, 1162–1173. doi: 10.1111/j.1365-2745.2008.01435.x

[B9] Gallego-Tévar B. Rubio-Casal A. E. de Cires A. Figueroa E. Grewell B. J. Castillo J. M.. (2018). Phenotypic plasticity of polyploid plant species promotes transgressive behaviour in their hybrids. AoB Plants. 10 (5). doi: 10.1093/aobpla/ply055 PMC6201833 30377487

[B10] Gastwirth J. L. Gel Y. R. Hui W. L. W. Lyubchich V. Miao W. Noguchi K.. (2020). ‘lawstat: Tools for biostatistics, public policy, and law,” in R package version 3.4.

[B11] Gómez J. M. Perfectti F. Armas C. Narbona E. González-Megías A. Navarro L.. (2020). Within-individual phenotypic plasticity in flowers fosters pollination niche shift. Nat. Commun. 11 (1). doi: 10.1038/s41467-020-17875-1 PMC7419554 32782255

[B12] Hiatt D. Flory S. L. (2020). Populations of a widespread invader and co‐occurring native species vary in phenotypic plasticity1. New Phytol. 225, 584–594. doi: 10.1111/nph.16225 31566739

[B13] Jiang X. Song Q. Ye W. Chen Z. J. (2021). Concerted genomic and epigenomic changes accompany stabilization of arabidopsis allopolyploids. Nat. Ecol. Evol. 5, 1382–1393. doi: 10.1038/s41559-021-01523-y 34413505 PMC8484014

[B14] Kakutani T. Jeddeloh J. A. Flowers S. K. Munakata K. Richards E. J. (1996). Developmental abnormalities and epimutations associated with DNA hypomethylation mutations. Proc. Natl. Acad. Sci. United States America. 93 (22), 12406–12411. doi: 10.1073/pnas.93.22.12406 PMC38004 8901594

[B15] Madlung A. Masuelli R. W. Watson B. Reynolds S. H. Davison J. Comai L.. (2002). Remodeling of DNA methylation and phenotypic and transcriptional changes in synthetic arabidopsis allotetraploids. Plant Physiol. 129 (2), 733–746. doi: 10.1104/pp.003095 12068115 PMC161697

[B16] Manning K. Tör M. Poole M. Hong Y. Thompson A. J. King G. J.. (2006). A naturally occurring epigenetic mutation in a gene encoding an SBP-box transcription factor inhibits tomato fruit ripening. Nat. Genet. 38 (8), 948–952. doi: 10.1038/ng1841 16832354

[B17] March-Salas M. Fandos G. Fitze P. S. (2021). Effects of intrinsic environmental predictability on intra-individual and intra-population variability of plant reproductive traits and eco-evolutionary consequences. Ann. Bot. 127 (4), 413–423. doi: 10.1093/aob/mcaa096 32421780 PMC7988524

[B18] Noguchi K. Gel Y. R. (2010). Combination of levene-type tests and a finite-intersection method for testing equality of variances against ordered alternatives. J. Nonparametr. Stat. 22 (7), 897–913. doi: 10.1080/10485251003698505

[B19] Novikova P. Y. Tsuchimatsu T. Simon S. Nizhynska V. Voronin V. Burns R.. (2017a). Genome sequencing reveals the origin of the allotetraploid arabidopsis suecica. Mol. Biol. Evol. 34 (4), 957–968. doi: 10.1093/molbev/msw299 28087777 PMC5400380

[B20] Novikova P. Y. Tsuchimatsu T. Simon S. Nizhynska V. Voronin V. Burns R.. (2017b). Genome sequencing reveals the origin of the allotetraploid Arabidopsis suecica. Mol. Biol. Evol. 34, msw299. doi: 10.1093/molbev/msw299 PMC5400380 28087777

[B21] Paape T. Briskine R. V. Halstead-Nussloch G. Lischer H. E.L. Shimizu-Inatsugi R. Hatakeyama M.. (2018). Patterns of polymorphism and selection in the subgenomes of the allopolyploid Arabidopsis kamchatica. Nat. Commun. 9 (1), 3909. doi: 10.1038/s41467-018-06108-1 30254374 PMC6156220

[B22] Paterson A. H. (2005). Polyploidy, evolutionary opportunity, and crop adaptation. Genetica (Springer) 123, 191–196. doi: 10.1007/s10709-003-2742-0 15881691

[B23] Potente G. Léveillé-Bourret É. Yousefi N. Choudhury R. R. Keller B. Diop S. I.. (2022). Comparative genomics elucidates the origin of a supergene controlling floral heteromorphism. Mol. Biol. Evol. 39 (2). doi: 10.1093/molbev/msac035 PMC8859637 35143659

[B24] R Core Team (2020). R: A language and environment for statistical computing (Vienna, Austria: R Foundation for Statistical Computing). Available at: https://www.r-project.org/.

[B25] Rice A. Šmarda P. Novosolov M. Drori M. Glick L. Sabath N.. (2019). The global biogeography of polyploid plants. Nat. Ecol. Evol. 3 (2), 265–273. doi: 10.1038/s41559-018-0787-9 30697006

[B26] Schmid M. W. Heichinger C. Coman Schmid D. Guthörl D. Gagliardini V. Bruggmann R.. (2018). Contribution of epigenetic variation to adaptation in arabidopsis. Nat. Commun. 9 (1), 1–12. doi: 10.1038/s41467-018-06932-5 30361538 PMC6202389

[B27] Seymour D. K. Becker C. (2017). The causes and consequences of DNA methylome variation in plants. Curr. Opin. Plant Biol. 36, 56–63. doi: 10.1016/j.pbi.2017.01.005 28226269

[B28] Shimizu-Inatsugi K. K. (2022). Robustness and the generalist niche of polyploid species: Genome shock or gradual evolution? Curr. Opin. Plant Biol. 69, 102292. doi: 10.1016/j.pbi.2022.102292 36063635

[B29] Shimizu-Inatsugi R. LihovÁ J. Iwanaga H. Kudoh H. Marhold K. Savolainen O.. (2009). The allopolyploid arabidopsis kamchatica originated from multiple individuals of Arabidopsis lyrata and Arabidopsis halleri. Mol. Ecol. 18 (19), 4024–4048. doi: 10.1111/j.1365-294X.2009.04329.x 19754506

[B30] Tsuchimatsu T. Kaiser P. Yew C.-L. Bachelier J. B. Shimizu K. K.. (2012). Recent loss of self-incompatibility by degradation of the Male component in allotetraploid arabidopsis kamchatica. PloS Genet. 8 (7), e1002838. doi: 10.1371/journal.pgen.1002838 22844253 PMC3405996

[B31] Van de Peer Y. Mizrachi E. Marchal K. (2017). The evolutionary significance of polyploidy. Nat. Rev. Genet. 18 (7), 411–424. doi: 10.1038/nrg.2017.26 28502977

[B32] Wei N. Cronn R. Liston A. and Ashman T. L. (2019). Functional trait divergence and trait plasticity confer polyploid advantage in heterogeneous environments. New Phytologist. 221 (4), 2286–2297. doi: 10.1111/nph.15508 30281801 PMC6587808

[B33] Westerband A. C. Funk J. L. Barton K. E. (2021). Intraspecific trait variation in plants: A renewed focus on its role in ecological processes. Ann. Bot. 127, 397–410. doi: 10.1093/aob/mcab011 33507251 PMC7988520

[B34] Yuan J. Jiao W. Liu Y. Ye W. Wang X. Liu B.. (2020). Dynamic and reversible DNA methylation changes induced by genome separation and merger of polyploid wheat. BMC Biol. 18 (1), 171. doi: 10.1186/s12915-020-00909-x 33218336 PMC7679994

